# Identification of Vancomycin Resistance in Methicillin-resistant *Staphylococcus aureus* in two macaque species and decolonization and long-term prevention of recolonization in Cynomolgus Macaques (*Macaca fascicularis*)

**DOI:** 10.3389/fimmu.2023.1244637

**Published:** 2023-08-22

**Authors:** Rachele M. Bochart, Kimberly Armantrout, Hugh Crank, Rachael Tonelli, Christine Shriver-Munsch, Tonya Swanson, Miranda Fischer, Helen Wu, Michael Axthelm, Jonah Sacha, Jeremy V. Smedley

**Affiliations:** ^1^ Vaccine and Gene Therapy Institute, Oregon Health and Science University, Beaverton, OR, United States; ^2^ Division of Pathobiology and Immunology, Oregon National Primate Research Center, Vaccine and Gene Therapy Institute, Oregon Health and Science University, Beaverton, OR, United States

**Keywords:** antibiotic resistance, methicillin-resistant *Staphylococcus aureus*, vancomycin- resistant *Staphylococcus aureus*, rhesus macaques, cynomolgus macaques, occupational safety, decolonization, mupirocin

## Abstract

Methicillin-resistant *Staphylococcus aureus* (MRSA) is a *S. aureus* strain with resistance to beta-lactam antibiotics, making it a global human and veterinary health concern. Specifically, immunosuppressed patients have a remarkably higher risk of clinical MRSA infections with significantly increased rates of prolonged clinical recovery, morbidity, and mortality. The current treatment of choice for MRSA is vancomycin. Importantly, we report the first known vancomycin-resistant *S. aureus* (VRSA) carriers in a cohort of Mauritian cynomolgus macaques (CM) imported to the Oregon National Primate Research Center (ONPRC), with a MRSA carrier rate of 76.9% (10/13 animals). All MRSA isolates also demonstrated resistance to vancomycin with prevalence of vancomycin-intermediate *Staphylococcus aureus* (VISA) at 30% (3/10 MRSA-positive CMs) and VRSA at 70% (7/10 MRSA-positive CMs). Additionally, we identified VRSA in a rhesus macaque (RM) housed within the same room as the VRSA-positive CMs and identified a MRSA/VISA carrier rate of 18.8% in RMs (3/16 positive for both MRSA and VISA) in unexposed recently assigned animals directly from the ONPRC RM breeding colony. Considering that the MRSA and VRSA/VISA-positive CMs future study aims included significant immunosuppression, MRSA/VRSA/VISA decolonization treatment and expanded “MRSA-free” practices were employed to maintain this status. We report the first controlled study using in-depth analyses with appropriate diagnostic serial testing to definitively show an MRSA decolonization therapy (90% success rate) and expanded barrier practice techniques to successfully prevent recolonization (100%) of a cohort of CMs MRSA-free (up to 529 days with a total of 4,806 MRSA-free NHP days).

## 1 Introduction

Methicillin-resistant *Staphylococcus aureus* (MRSA) has been found in numerous captive and non-captive colonies of macaques (1–7) and is both increasingly prevalent in community settings and a major nosocomial pathogen in human hospitals. Macaques are often imported to the US from regions with high MRSA prevalence in human and nonhuman primate populations, which may be in part from zoonotic and zooanthroponotic transmission of MRSA among other factors (1, 8–10). MRSA is transmitted through direct contact and fomites with colonization occurring in moist areas, primarily the nares, but additional sites can include skin and other mucosal surfaces, especially in the inguinal, axillary, genital, and rectal areas. MRSA colonized macaques are both an occupational risk, with documented suspected cases of zoonotic transmission to exposed staff (11, 12), and a risk to their clinical health, as nasal *S. aureus* carriers have more than double the rate of *S. aureus* infections compared to MRSA-free patients (13). This risk to macaques is further increased when they are employed in models that involve immunosuppression, surgery, and/or surgical implants, especially implants that result in chronic penetration of the skin, such as indwelling catheters or cranial implants (14). According to the CDC, there are 5 C’s that promote MRSA transmission in people, and they are “Crowding, frequent skin-to-skin Contact, Compromised skin, Contaminated items and surfaces, and lack of Cleanliness” (15), and all of these factors can exist in macaque facilities. Macaques are often housed at primate centers in large numbers, typically socially housed resulting in frequent contact and often acquire injuries that break the skin from typical intraspecies social interactions. They are often worked with in procedure rooms with shared equipment, and groups of animals of the same status/experimental group are often handled simultaneously with limited sanitation between animals for routine procedures such as phlebotomy.

At the ONPRC, we have been developing and optimizing a model of allogeneic hematopoietic stem cell transplantation (HSCT) using imported Mauritian cynomolgus macaques (CMs) (16–18). This model has been highly informative for the mechanisms and dynamics of simian immunodeficiency virus (SIV) reservoir eradication; however, it requires significant immunosuppression, surgical sampling of potential tissue reservoir sites (19, 20), and chronic indwelling catheters to permit frequent sampling and drug administration, all of which lead to increased susceptibility to MRSA infection. We lost one of these valuable animals to fulminant MRSA septicemia and have more where MRSA was present and a potential contributing factor to morbidity and mortality in this model. We thus undertook an assessment of baseline rates of MRSA colonization and antibiotic resistance profiles of MRSA isolates, and determined that the CMs at the ONPRC had a high prevalence of highly antibiotic resistant MRSA, including the presence of vancomycin-intermediate *S. aureus* (VISA) and vancomycin-resistant *S. aureus* (VRSA), which has not been previously documented in macaques. Additionally, we identified VRSA in a rhesus macaque (RM) housed with the VRSA-positive CMs and identified a MRSA/VISA carrier rate of 18.8% in a room of RMs (3/16 positive for both MRSA and VISA) unexposed to CMs and recently assigned coming directly from the ONPRC RM breeding colony. These findings are concerning, considering that there have been only 14 documented human cases in the United States of VRSA, with VISA reported at a higher rate, although both have remained susceptible to other antibiotics (21).

Following up on encouraging results obtained at the WaNPRC, where attempts at decolonization (eradication of MRSA so the animal is no longer colonized as evidenced by negative MRSA culture results) of a primarily pigtail macaque population resulted in elimination of MRSA from 90% of the macaques at a 4-week post-treatment time point (11), we attempted a similar decolonization regimen in this colony of CMs. Additionally, we developed and deployed barrier practices based on our successful gastrointestinal pathogen-free (GPF) practices (22), which are used to maintain animals free of pathogenic enteric bacteria. Here, we present the methodology and results of a MRSA decolonization therapy, including barrier practices, which were employed to eradicate MRSA and maintain animals MRSA free for an extended period. Rates of decolonization matched those from the WaNPRC and published rates in people (23). However, most striking was the ability to successfully prevent recolonization in all of the decolonized animals for up to 17 months, demonstrating through the frequent use of appropriate diagnostics for the first time that MRSA recolonization can be prevented with appropriate barrier practices for a prolonged (>17 months) period of time.

## 2 Results

**Figure 1:**
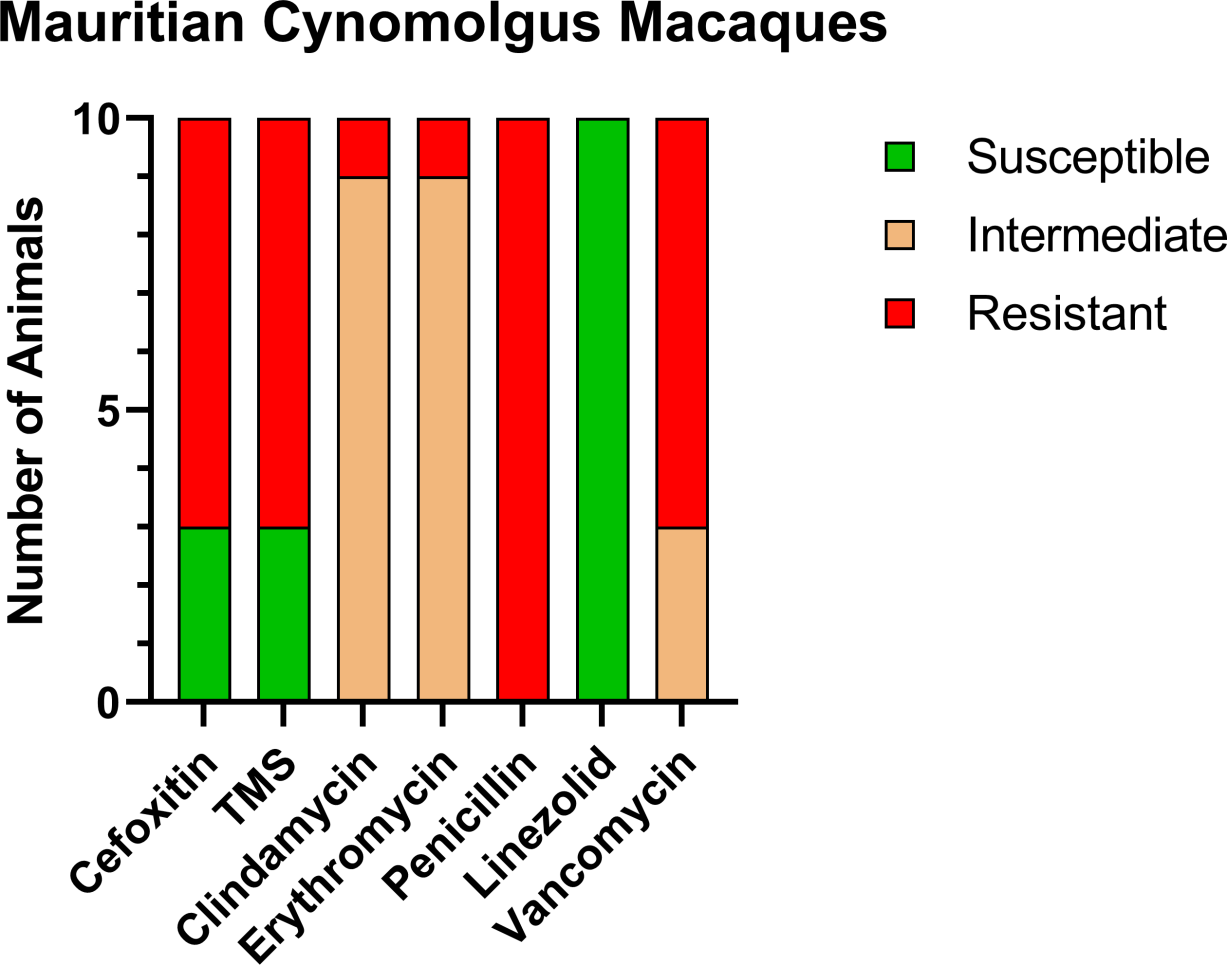
Antibiotic sensitivity results for the 10 MRSA-positive CM prior to decolonization therapy show a high rate of resistance profiles including VRSA (7/10) and VISA (3/10). The number of animals (y-axis) and each antibiotic resistance profile (x-axis) are represented by a single vertically stacked bar plot; antibiotic susceptible (green), intermediate (orange), and resistant (red).

Of the CM enrolled in this study, 10/13 CMs (76.9%) were positive for MRSA on nasal culture prior to treatment with variable patterns of resistance (**Figure 1**). MRSA isolates were resistant to cefoxitin in 7/10, trimethoprim-sulfamethoxazole (TMS) in 7/10, clindamycin in 1/10, erythromycin in 1/10, penicillin in 10/10, and vancomycin in 7/10. In addition, MRSA isolates showed intermediate resistance to clindamycin in 9/10, erythromycin in 9/10, and vancomycin in 3/10.

**Figure 2:**
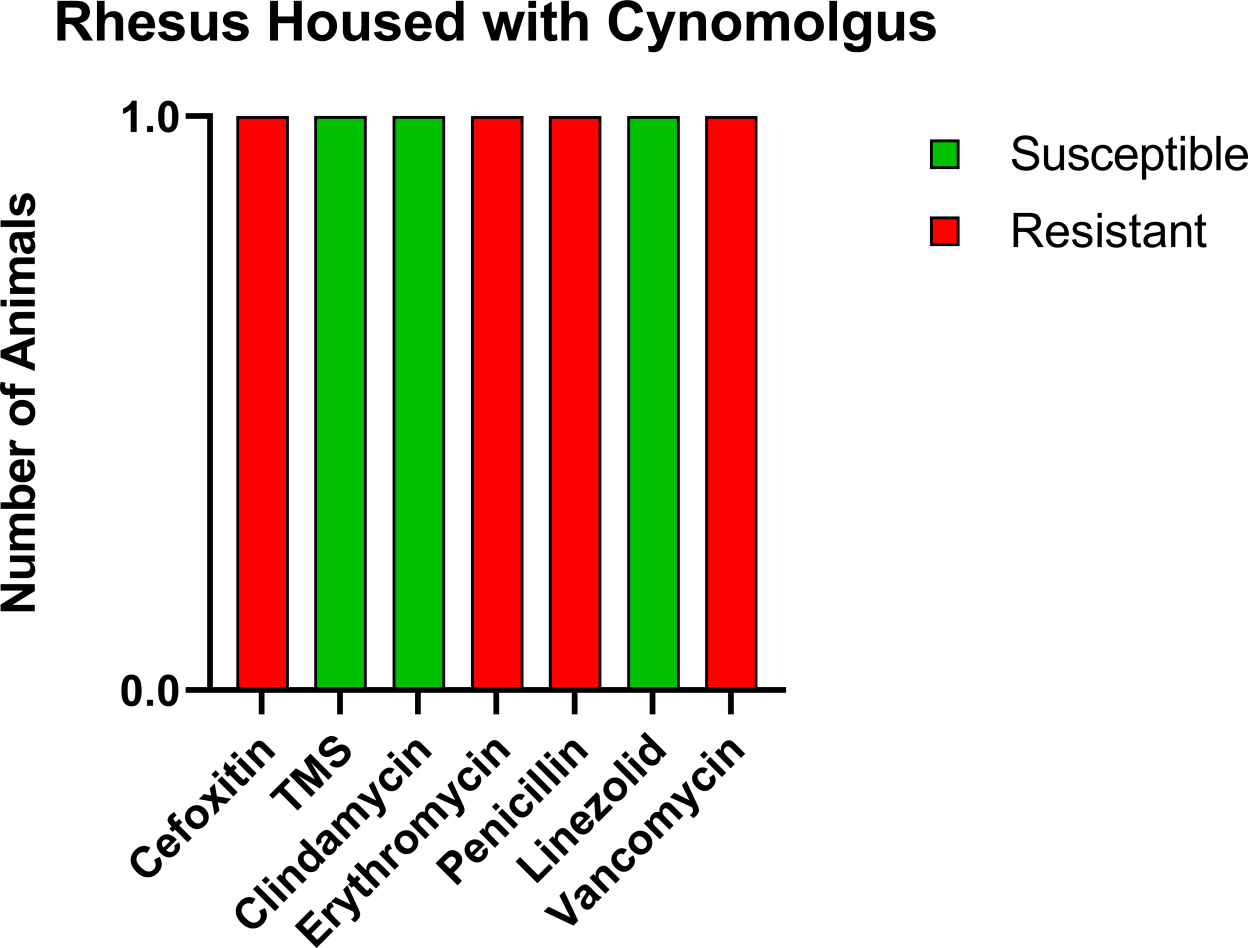
Similar antibiotic sensitivity patterns as the CMs were present for the one MRSA-positive RM that was housed within the same room, including VRSA. Each antibiotic resistance profile (x-axis) is represented by a bar plot for the single MRSA-positive RM (y-axis); antibiotic susceptible (green), intermediate (orange), and resistant (red).

Prior to the study, the CMs were housed with six rhesus macaques (RMs) born at ONPRC. To determine if transmission was occurring between the CMs and RMs housed in the same room but not in the same cages, we tested these six RMs. Only one of six (16.7%) was positive for MRSA, and the resistance profile was similar to what was seen in the CMs including complete resistance to cefoxitin, erythromycin, penicillin, and vancomycin, making the RM isolate VRSA as well as MRSA (**Figure 2**).

**Figure 3:**
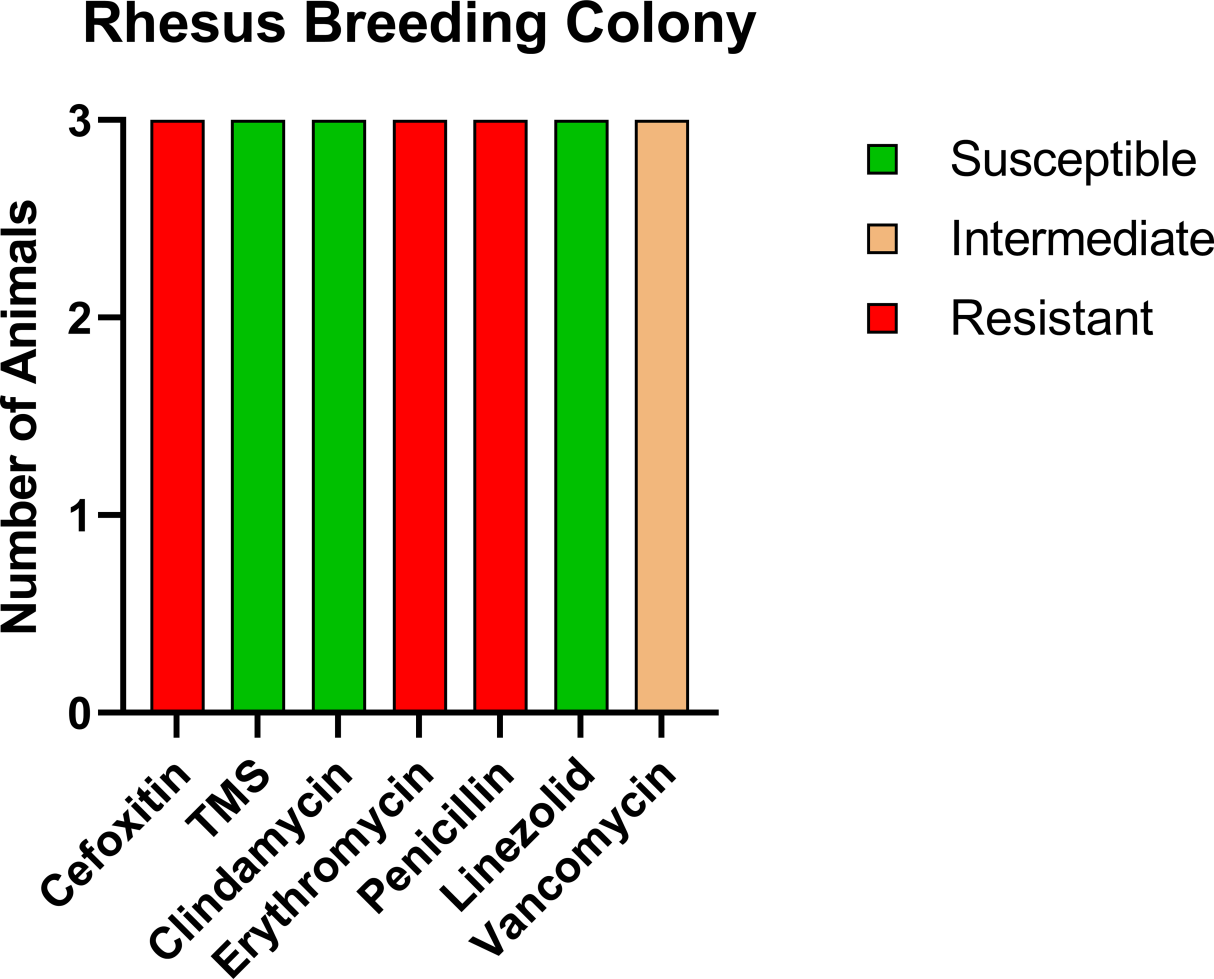
Antibiotic sensitivity results for the three MRSA-positive RMs with no history of prior exposure to CMs differed for the antibiotic sensitivity profile, with high prevalence of VISA (100%, 3/3 RMs) and no evidence of VRSA. The number of animals (y-axis) and each antibiotic resistance profile (x-axis) are represented by a single vertically stacked bar plot; antibiotic susceptible (green), intermediate (orange), and resistant (red).

For comparison, we tested a room of RMs that had been recently moved in from the breeding colony with no exposure to CMs. Of the 16 animals tested, three were positive for MRSA (18.8%); however, the profile of resistance was distinct from both the CMs and the RM exposed to CMs (**Figure 3**), with only intermediate resistance to vancomycin (VISA). VRSA and VISA are thought to be due to distinct genetic changes (24), indicating that the RM with exposure to CMs potentially contracted VRSA from the CMs.

MRSA decolonization was administered for all 13 CMs, which included a therapeutic regimen of medicated chlorhexidine baths and 0.2% chlorhexidine dentition/oral cavity cleaning (SID for 5 days); antimicrobial ophthalmic ointment applied to both eyes and 2% mupirocin ointment administered to the urogenital areas (BID for 5 days, followed by SID for 2 days); decolonization treatment for the first group of seven animals received erythromycin 50 mg/kg BID for 10 days.

CMs were tested at 1, 2, and 4 weeks post-decolonization, and 12/13 tested negative at all three time points. The remaining animal that tested positive at 1-week post-decolonization was relocated to a MRSA-positive room and eliminated from further follow-up. Thus, 9/10 previously positive animals were decolonized at the 4-week point representing a 90% success rate for the decolonization protocol. During the entire post-decolonization period, the CMs were housed utilizing the MRSA-free barrier practices, over 17 months (Group 1) and 13 months (Group 2). Subsequent rounds of testing of the remaining 12 animals occurred at 1–3-month intervals totaling 7–16 tests/animal (total 107 nasal cultures) with all animals testing negative at each time point. This represents 100% successful prevention of recolonization or an acquisition rate of 0/4,806 NHP days.

Other MRSA-positive CMs were housed in the same section of the building (ASB3) during the follow-up period. Additionally, MRSA-positive CMs and RMs go to surgery, procedure rooms, and in vehicles, so there was constant potential for exposure of MRSA-free animals throughout the study period.

## 3 Discussion

We identified an extremely high prevalence of MRSA (77%), VRSA (54%), and VISA (23%) in a small colony of Mauritian cynomolgus macaques residing at the ONPRC. Limited data were available for MRSA status at the time of quarantine; only 8/13 CMs were tested with 1/8 positive, and some of the negative CMs were received with shipments of MRSA-positive animals. As no antibiotic sensitivity testing was performed at this time, it is unclear if the high rates of VRSA and VISA represent their status upon arrival, or if these were transferred between animals either in quarantine (only CMs) or subsequently in standard ABSL2 housing (CMs housed with RMs) at the ONPRC prior to enrollment in this project. Published rates of comparable nasal testing in human hospitals are much lower with typical rates of colonization ranging between 7.2% and 16.3% for MRSA and 0% for VRSA/VISA (25, 26), and even in clinical samples, rates are typically much lower for VRSA/VISA infection (27, 28). To try to determine the origin of the VRSA/VISA, we screened both RMs that had been housed with the CMs and RMs directly from the breeding colony with no exposure to CMs. The resistance profile in the RMs with no exposure to CMs only documented VISA and not VRSA, and the molecular mechanisms of drug resistance are thought to be different for VRSA and VISA (29). Thus, it is likely that the VRSA came with the CMs and was transferred to the RM housed with them, whose isolate was also VRSA. Unfortunately, we did not retain samples and were thus unable to sequence the isolates, which would have provided definitive evidence of both the source and transmission. However, given the apparent transmission of VRSA from CMs to RMs housed in the same room, this appears to be a risk, and housing of different species, especially from different sources, should be considered to be a transmission risk.

The high prevalence of MRSA, VRSA, and VISA in the CMs presents a clinical health and occupational risk to laboratory animal staff. However, in this case, the CMs were also part of a project that involved significant immunosuppression, surgically implanted indwelling catheters, surgical biopsies, and transportation to a human healthcare facility, increasing their risk for zoonotic transmission and for clinical sequelae with one confirmed fatal septicemia and several additional cases where MRSA potentially contributed to the morbidity and mortality associated with this model. The animals enrolled in this project were thus selected for decolonization using a combination of mupirocin ointment in the nares, and on the genital mucosa, and chlorhexidine oral and topical treatments. Group 1 was additionally treated with oral erythromycin based on the sensitivity profile of the MRSA; however, as the rate for this group was no different from prior publications without the oral treatment, this was discontinued for Group 2. In total, this resulted in elimination in 9/10 animals that tested positive prior to treatment, with one animal removed from the room/study when it failed to decolonize on the week 1 post-decolonization sample. Three additional animals were negative prior to the treatment but were treated as well to ensure that they were not recently exposed, as they had been housed in the same room as the animals testing positive. These rates of decolonization are highly comparable to similar protocols in people where 90% were also decolonized 1 week after treatment with mupirocin (30).

However, the exciting difference in our study is that follow-up testing involving up to 15 additional nasal cultures per animal demonstrated that all animals remained MRSA free up to 17 months (**Supplementary Table S1**), whereas only approximately 60% of decolonized people remained decolonized when rechecked between 2 weeks and 12 months post-treatment (30). While the numbers are small (12 CMs/4,806 NHP days), to the best of our knowledge, this represents the first 100% successful prevention of MRSA recolonization post-decolonization reported in the human or macaque literature and demonstrates that with appropriate PPE, disinfection, and barrier practices, it is possible to keep MRSA out of a room of indoor-housed CMs even when other rooms in the same section of the building contain animals known to be positive. The practices employed in this study and described in detail in the methods were successful despite sharing procedure rooms, surgical suites, and transportation to human healthcare facilities using shared vehicles and the potential for exposure in the healthcare facility itself, all of which represent important points of potential exposure. Thus, appropriate disinfection, barrier, and PPE practices, when employed consistently, can reliably prevent the spread of MRSA even in high-risk settings.

The advantage of working with macaques in an ABSL2 facility is that all surfaces are sanitizable and they do not have fabrics or other types of materials that are more difficult to decontaminate. However, this study is a proof of concept that MRSA colonization/recolonization can be prevented, and it is therefore possible that if additional practices were employed to address factors such as clothing and bedding present in patient’s environments, similar practices could be equally effective in preventing MRSA in hospital settings (31). Improvements in disinfection have been shown to dramatically decrease recolonization rates (32), and taking these a step further should lead to even further reductions based on our results.

For macaque colonies, this study provides good rationale for testing animals for MRSA, both to determine prevalence within colonies, prior to transport, and/or in quarantine to prevent the introduction of MRSA and/or different strains of MRSA that may have different antibiotic resistance profiles or virulence factors that could recombine with strains present in the recipient colony to produce worse outcomes (11). The high rates of VRSA and VISA also present a rationale to test animals entering the US and animals received from US vendors to ensure that these organisms are not introduced into macaque colonies and become an occupational health hazard and in turn a potential community health concern. These are both in keeping with the Guide for the Care and Use of Laboratory Animals recommendation to test animals for pathogens in quarantine and that “Intraspecies separation may be essential when animals obtained from multiple sites or sources, either commercial or institutional, differ in pathogen status… (33)”.

Finally, the presence of MRSA, VRSA, and VISA in the ONPRC rhesus colony in addition to the CMs suggests that NHP veterinarians should highly limit the use of indiscriminate and unnecessary prophylactic antibiotic use to prevent further emergence of antimicrobial resistance in these valuable breeding and research colonies (34). Vancomycin-intermediate resistance is present despite the fact that vancomycin has not been used in the ONPRC’s rhesus breeding colony as a treatment in decades, and its use in RMs at the ONPRC in the past >20 years has been limited to two research RMs associated with surgical or implant related infections. Previously, we reported the potential for some common antibiotics to have significant impact on outcomes in models such as SIV when prescribed to primates during infectious disease studies (35), indicating that use, not just in breeders but also in study animals during the course of the experiment, should be minimized. Thus, it is best to ensure animals engaged in infectious disease studies are free of pathogens that might increase the likelihood that they will become clinically ill and/or require antibiotic treatment during the experimental phase (22) as we did here by eliminating MRSA, VRSA, and VISA from CMs on high-risk projects.

In conclusion, we documented high rates of MRSA, VRSA, and VISA in imported CMs, MRSA, and VISA in ONPRC rhesus from the breeding colony and the likely transmission of VRSA from the CMs to an RM housed in the same room. We were able to successfully decolonize 90% of MRSA-carrier CMs using a combination of mupirocin ointment and chlorhexidine oral and topical treatments. We were also able to demonstrate for the first time that it is possible to maintain decolonized animals free of MRSA for extended periods, in this case maintaining 100% MRSA-free for the entire duration of the study (up to 17 months) by using expanded barrier practices.

## 4 Materials and methods

### 4.1 Animal cohort

A total of 13 Mauritius-origin cynomolgus macaques (five females/eight males) (**Table 1**) between 3 and 9 years of age were used in accordance to the institutional policies at the Oregon National Primate Research Center, an AAALAC-accredited facility that abides by the USDA Animal Welfare Regulations (36), the Guide for the Care and Use of Laboratory Animals (33), and the Public Health Service Policy on Humane Care and Use of Laboratory Animals (37). All animals were imported to ONPRC within the years of 2016–2021 from either PrimeGen, LLC, Mannheimer Foundation, Inc., or Worldwide Primates, Inc., and inhabited standardized housing prior to study start and were individually or socially housed during the study in accordance with the IACUC protocol and facility practices. Throughout the study, all animals were uniformly fed Purina LabDiet 5000, 5045, or 5047 or TAD Primate Diet no. 5LOP Test Diet (Purina Mills International, St. Louis, MO) daily nutritional enrichment items (grains, fruits, or vegetables), *ad libitum* access to water. In Group 1, three of six animals were humanely euthanized for HSCT protocol reasons (unrelated to the MRSA decolonization study) prior to the 529 days (CM2, 225 days; CM4, 298 days; and CM3, 223 days); for Group 2, all six animals that remained in the study for the entirety of the study duration.

**Table 1:** CM demographics and days MRSA-free post-decolonization treatment.

Animal	Sex	Age	Post-MRSA treatment (MRSA-free days)
CM1	Female	7.1	529
CM2	Female	9.55	225*
CM3	Male	5.82	223*
CM4	Male	4.93	298*
CM5	Female	8.35	529
CM6	Male	7.29	529
CM7	Female	6.93	N/A^1^
CM8	Male	4.18	411
CM9	Male	4.23	411
CM10	Male	6.64	411
CM11	Female	3.6	411
CM12	Male	7.05	411
CM13	Male	6.27	418
**Total MRSA-free days**			**4,806**

^*^CMs humanely euthanized for HSCT protocol reasons (unrelated to MRSA decolonization study).

^1^Removed from the room/study when it failed MRSA decolonization.

A total of 16 ONPRC-captive born rhesus macaques (4 females/12 males) between 2.5 and 5 years of age received pre-study MRSA tests, and results were reported in this publication, with no history of housing near cynomolgus macaques. An additional six ONPRC-captive born rhesus macaques (five female/one male) between 3 and 6 years of age were MRSA tested due to sharing a room with cynomolgus macaques that were MRSA positive. All of these animals were utilized as described above to the ONPRC policies and regulations, and they were fed and socially housed in accordance to institutional and IACUC policies.

### 4.2 MRSA decolonization regimen

All 13 CMs received MRSA decolonization treatment. Animals received either ketamine (Ketathesia™, Henry Schein Animal Health)/dexmedetomidine hydrochloride (Dexmedesed™, Dechra, Overland Park, KS) (reversed by atipamezole hydrochloride (Antisedan ®, Zoetis, Kalamazoo, MI; Revertidine™; Modern Veterinary Therapeutics, LLC, Miami, FL) or ketamine for sedation events for decolonization treatment. The treatment included administration of antimicrobial ophthalmic ointment applied to both eyes (Vetericyn Plus ®, Innovacyn Inc., Rialto, CA) for (5 days BID, followed by 2 days SID) 7 days total; a thin layer of 2% mupirocin ointment (Taro Pharmaceuticals USA, Inc., Hawthorne, NY) was administered to the following areas: preputial area of males (1 mL), intravaginally for females (1 mL), bilateral nasal cavities (males, 1 mL total/female, 0.6 mL total); twice daily for 5 days, then once daily for 2 days (7 total days). A medicated bath was administered for 5 days using TrizChlor™4 (TrizChlor™4, Dechra, Overland Park, KS) shampoo utilized as directed on the label, the medicated shampoo was fully rinsed, and the animal was dried off by a sterile towel. Diluted chlorhexidine 0.2% (chlorhexidine solution 2%, Covetrus, Tualatin, OR) on sterile gauze was used to carefully wipe the oral cavity/buccal mucosa and oropharynx and used to brush dentition with sterile toothbrushes for 5 days. For both groups, midweek of the decolonization treatments, new feed cart/PPE station, consumables, and enrichment devices were utilized; a full cage change with deep cleaning of walls/floors/working space outside the room was performed; and the working space outside the room was demarcated by a partitioned taped off line, and MRSA-free practices were employed. The first group of seven animals received erythromycin 50 mg/kg BID for 10 days. The second group of six animals and the second round of MRSA decolonization for a single CM (CM1) that had social access to the one animal (CM7) that failed decolonization did not receive erythromycin to minimize antibiotic use and due to the rate of decolonization in the first group not being different from the rate of MRSA decolonization at another facility (11) where systemic antibiotics were not used. Animals received three serial negative tests prior to designating them as MRSA free.

### 4.3 MRSA testing

Animals were sedated with ketamine, and the nasal cavity/nasopharynx was swabbed bilaterally using sterile cotton swabs (BD BBL CultureSwab plus Liquid Stuart Medium or BD BBL CultureSwab plus Amies Medium, Becton Dickinson, Sparks, MD, US) and were sent to VRL Diagnostics (San Antonio, USA) for NHP MRSA testing. Samples were determined to be MRSA positive by confirmatory standard testing at VRL Diagnostics and by the use of spectra MRSA medium; positive samples had an antibiotic sensitivity profile performed following the Clinical and Laboratory Standards Institute (CLSI) Performance Standards for Antimicrobial Susceptibility Testing (38); for animals with multiple positive tests, the most resistant antibiotic profile is reported.

### 4.4 Description of practices to maintain MRSA-free status

Enhanced nonhuman primate biosecurity practices were used to exclude MRSA from this high-risk CM research group; practices were similar to those utilized for our eSPF and gastrointestinal pathogen-free practices (22). The MRSA-free room was designated as a primary entry room and located in the animal facility to limit access and reduce the risk of MRSA contamination. The multipurpose MRSA-free work area was demarcated off by a taped boundary, which included a MRSA-free PPE station, husbandry, clinical, research work surfaces, and storage. Prior to entering the MRSA-free boundary, the staff don new MRSA-free designated personal protective equipment (Tyvek suits, gloves, face shields, masks, hair bonnets, and boot covers). All supplies and equipment that are utilized for the decolonization process, or for husbandry, research, surgical, or clinical use for these animals undergo either vaporized hydrogen peroxide, ethylene oxide, autoclave sterilizations, or gross decontamination utilizing generous amounts of disinfectants with correct contact time that are known to disinfect MRSA, and when possible MRSA-free animals underwent planned procedures prior to other NHPs. All consumables and non-consumables used for study animals were new and/or appropriately disinfected prior to distribution and, when possible, separately stored from other animal areas. NHP housing equipment are cage washed and disinfected as they enter into MRSA-free boundary. MRSA tests were taken serially to ensure that animals remained MRSA free for the duration of this study. Additionally, animals were tested to ensure that they remain MRSA free prior to immunosuppression for hematopoietic stem cell transplant and post-transplant, transportation to a satellite facility with a high-risk of human MRSA, and at higher frequencies for animals that receive procedures in shared surgical suites with MRSA-positive animals.

### 4.5 Data extraction and statistical analyses

The ONPRC’s electronic animal health record system was utilized to extract demographic, research, and clinical data for this study, and VRL Diagnostic’s MRSA and antibiotic-resistant profile reports. Figures of the data were made by GraphPad Prism version 9.5.1.

## Data availability statement

The raw data supporting the conclusions of this article will be made available by the authors, without undue reservation.

## Ethics statement

The animal study was approved by Oregon Health and Science University. The study was conducted in accordance with the local legislation and institutional requirements.

## Author contributions

JVS: writing original draft, investigation, data curation, supervision, formal analysis, methodology, conceptualization, project administration, funding acquisition, and validation. RMB: writing original draft, investigation, data curation, supervision, formal analysis, methodology, conceptualization, and validation; HC: data curation, formal analysis, methodology, validation, and software. KA, CS-M, RT, TS, and MF: investigation, review, and editing manuscript. HW: methodology, conceptualization, and review and editing manuscript. JS: conceptualization, funding acquisition, project administration, methodology, and review and editing manuscript. MA: project administration, conceptualization, methodology, and review and editing manuscript. All authors listed contributed to the manuscript and approved it for publication.
