# Supplementary material for: Identification of Vancomycin Resistance in Methicillin-resistant Staphylococcus aureus in two macaque species and decolonization and long-term prevention of recolonization in Cynomolgus Macaques (Macaca fascicularis)
Source: Front Immunol. 2023 Aug 22;14:1244637. doi: 10.3389/fimmu.2023.1244637 (PMC10477669; doi:10.3389/fimmu.2023.1244637)
Supplement: Supplementary file 1 [file Table_1.docx]

**Supplemental Table 1. MRSA results prior- to and post-decolonization for the CM study cohort.**

| **Animal** | **Group** | **MRSA status prior to Decolonization** | **Antibiotic Sensitivity Profile prior to MRSA-decolonization^1^** | **Number of follow-up MRSA Tests Post-decolonization^2^** |
| --- | --- | --- | --- | --- |
| CM1 | 1 | Positive | Resistant: Cef, TMS, Pen Vanc | 16 |
|  |  |  | Intermediate: Clin, Ery |  |
|  |  |  | Susceptible: Lin |  |
| CM2 | 1 | Positive | Resistant: Cef, TMS, Pen | 7^*^ |
|  |  |  | Intermediate: Clin, Ery, Vanc |  |
|  |  |  | Susceptible: Lin |  |
| CM3 | 1 | Positive | Resistant: Cef, TMS, Pen, Vanc | 7^*^ |
|  |  |  | Intermediate: Clin, Ery |  |
|  |  |  | Susceptible: Lin |  |
| CM4 | 1 | Positive | Resistant: Cef, TMS, Clin, Ery, Pen | 8^*^ |
|  |  |  | Intermediate: Vanc |  |
|  |  |  | Susceptible: Lin |  |
| CM5 | 1 | Positive | Resistant: Cef, TMS, Pen | 10 |
|  |  |  | Intermediate: Clin, Ery, Vanc |  |
|  |  |  | Sensitive: Lin |  |
| CM6 | 1 | Positive | Resistant: Cef, TMS, Pen, Vanc | 14 |
|  |  |  | Intermediate: Clin, Ery |  |
|  |  |  | Sensitive: Lin |  |
| CM7 | 1 | Positive | Resistant: Cef, TMS, Pen, Vanc | n/a^3^ |
|  |  |  | Intermediate: Clin, Ery |  |
|  |  |  | Sensitive: Lin |  |
| CM8 | 2 | Negative | n/a | 7 |
| CM9 | 2 | Positive | Resistant: Pen, Vanc | 7 |
|  |  |  | Intermediate: Ery, Clin |  |
|  |  |  | Sensitive: Cef, TMS, Lin |  |
| CM10 | 2 | Negative | n/a | 7 |
| CM11 | 2 | Negative | n/a | 9 |
| CM12 | 2 | Positive | Resistant: Pen, Vanc | 7 |
|  |  |  | Intermediate: Clin, Ery |  |
|  |  |  | Sensitive: Cef, TMS, Lin |  |
| CM13 | 2 | Positive | Resistant: Pen, Vanc | 8 |
|  |  |  | Intermediate: Clin, Ery |  |
|  |  |  | Sensitive: Cef, TMS, Lin |  |

**Abbreviations:** Cef: Cefoxitin; TMS: Trimethoprim/Sulfamethoxazole; Clin: Clindamycin; Ery: Erythromycin; Pen: Penicillin; Lin: Linezolid; Vanc: Vancomycin; n/a: not applicable

**^1^** Results are from VRL Diagnostics; a commercial veterinary diagnostic laboratory specializing in nonhuman primates

^2^All MRSA results on post-decolonization follow-up testing were negative

**^3^** CM7 was removed from the room/study after failing MRSA decolonization

^*^ CMs humanely euthanized for HSCT protocol reasons (unrelated to MRSA decolonization study)
